# Seroepidemiology and risk factors of hepatitis C virus infection in East Azerbaijan, Iran: a population-based Azar Cohort study

**DOI:** 10.22088/cjim.10.3.326

**Published:** 2019

**Authors:** Ali Asghar Pouri, Morteza Ghojazadeh, Behrouz Pourasghari, Babak Baiaz, Fatemeh Soghra hamzavi, Mohammad Hossein Somi

**Affiliations:** 1 Liver and Gastrointestinal Diseases Research Center, Tabriz University of Medical Sciences, Tabriz, Iran; 2 Laboratory Department Imam Reza Hospital, Tabriz University of Medical Sciences, Tabriz, Iran

**Keywords:** Hepatitis C, Seroepidemiology, Azar Cohort, Iran

## Abstract

**Background:**

Hepatitis C virus (HCV) is a blood-borne virus. It is a major global public health problem and can cause both acute and chronic hepatitis. The aim of this study was to report the epidemiological features of HCV infection and risk factors based on the data from Azar Cohort, East Azerbaijan province, Iran.

**Methods:**

The population of this study comprised the people in the age range of 35-70 years from Azar Cohort, East Azerbaijan province, Iran. The study was conducted between 2015 and 2016. Based on cluster sampling, 4, 949 people were selected and invited to complete the questionnaire and perform the tests. Blood samples collected in this study were analyzed to detect the presence of antibodies against HCV using enzyme immunoassay (ELISA) Kit. The positive samples were re-tested by qualitative HCV-RNA polymerase chain reaction. All data were analyzed using SPSS version 19.0 software.

**Results:**

The mean age of the participants was 49.15±9.02 years. Of these participants, 54.3% (n=2686) were females. Seven people (0.14%) were detected as HCV positive and the highest frequency was seen in the age range of 40-50 (0.16%). There was a statistical significant relationship between history of hospitalization (P=0.02) and history of abnormal urine (P=0.01) with the frequency of HCV infection.

**Conclusion:**

The findings of this study indicated that the frequency of hepatitis C virus infection is 0.14% in the general population of Azar Cohort.

Hepatitis C is a liver disease caused by hepatitis C virus (HCV). Acute HCV infection is usually asymptomatic and about 35% of the infected people spontaneously clear the virus infection within 6 months without any treatment (1). Meanwhile, 60-80% of HCV patients develop chronic hepatitis infection and 20% are at risk for cirrhosis and liver cancer, and approximately 350,000 people die every year from the complications of hepatitis C disease (2). The prevalence of hepatitis C in the world is an average of 3%, and about 185 million people are infected with hepatitis C virus (3). Hepatitis C virus is the second leading cause of chronic liver disease and the most important cause of liver cancer and liver transplantation (4). In terms of geographical dispersion among Middle Eastern countries, the prevalence of hepatitis C virus is often high in Egypt (14.5%), Pakistan (4%), Saudi Arabia (1.8%) and Yemen (1.1%) (5). Iran has a low hepatitis C prevalence in the region. According to a survey conducted in Iran, the seroprevalence of hepatitis C infection was 0.6% and the prevalence of viremia was 0.4% (6); 186,500 people were infected with HCV (7) and almost 80% of the infected people were unaware of their disease (8). Unfortunately, the prevalence of this virus is increasing in the country, which necessitates careful analysis of this disease with the aim of taking the related health decisions (9).

The groups that are at high risk of HCV infection include hemophilias, thalassemic and hemodialysis patients and intravenous drugs addicts. These people can cause the infection to be transmitted to healthy people (6). According to the previous reports, the prevalence of hepatitis C virus in hemophiliacs, thalassemic patients, hemodialysis patients and intravenous drugs addicts is more than 40%, more than 20%, 13 to 18% and 11 to 52%, respectively (10, 11). The routes of transmission of hepatitis C infection are intravenous drug use, infected medical equipment, tattooing, needle stick, hemodialysis, infected blood and blood product, unsafe sexual activity and organ transplantation (9).

In the past, the most common way of hepatitis C transmission was blood transfusion, but after screening the blood donors since 1996, the predominant pathway of dissemination has been reported nosocomially (12). HCV infection is the main cause of mortality and disability and imposes financial costs on the communities and it is one of the major health issues in the world (13). There is no vaccine for hepatitis C, so early diagnosis can prevent the health problems caused by the hepatitis C virus infection. The risk of transmission of infectious diseases through blood transfusions is measurable by checking and analyzing the donors' information, screening methods and the incidence of serological markers of infectious diseases. Since the number of demographic studies carried out in East Azerbaijan province is limited and previous studies have merely investigated some certain groups of the community, and considering the impact of these infections on mortality rate, the cost of treatment and other socially harmful effects, this study was designed to determine the frequency and risk factors of hepatitis C virus transmission in this area.

## Methods

This project was part of Azar Cohort study in Shabestar, East Azerbaijan province (North West of Iran) and is part of large Persian cohort study (Prospective Epidemiological Research Studies of Iranian Adults) (14, 15). This cross-sectional study consisted of 5,000 males and females ranging from 35 to 70 were included between 2015 and 2016. The method of sampling was cluster sampling, including 50 clusters (100 subjects in each) from the mentioned county. In this cohort study, the first house on the right of the nearest street to the center of Azar Cohort was selected as the first household for each cluster randomly. After explaining the stages of the research for the included individuals, they were invited to the head office of the study for evaluation. Out of the 5,000 subjects, 51 people refused to get involved or were unable to participate and were excluded from the study, leaving a total of 4,949 individuals as the final sample.

The inclusion criteria were: residence in that area for more than a year, Iranian nationality, ability to answer to the questions and willingness to participate in the study. After starting the conduct of the research project, three separate groups were formed for interview, taking blood sample and laboratory analysis. A written and informed consent was obtained from the subjects prior to data collection. Then, a questionnaire containing demographic characteristics and risk factors for hepatitis such as age, sex, residence, level of education, marital status, history of blood transfusion, surgery, hospitalization, dental procedures, fatty liver, family history of hepatitis, traveling abroad, drug abuse, smoking and alcohol use was completed for all the eligible participants through face-to-face interviews. From each person, 10 ^cc^ of blood sample was taken. The blood samples were centrifuged, and the serums were separated and stored in micro tubes at -80 °C. The samples collected in this study were used for the detection of the presence of antibodies against HCV using enzyme immunoassay (ELISA) Kit, Anti HCV Ab (PISHTAZTEB, Tehran, Iran). Positive samples were re-tested by qualitative HCV-RNA polymerase chain reaction using HCV RQ Kit, produced by Novin Gene, Iran. 

The data were entered into SPSS.19 software. The qualitative data were analyzed using Chi-Square or Fisher's exact test with crude odds ratio (95%CI). The P-value less than 0.05 was considered as statistically significant.

## Results

Out of the total of 4,949 subjects, 2,686 individuals were females (54.3%) with an average age of 49.15±9.02 years (age range 35-70 years). Most participants were between 40-50 years of age (36.9%), married (93.4%) and non-employed (50.6%). Table 1 shows the demographic data of the participants in this HCV study. From among all the participants, 7 cases were identified as HCV Ab-positive by ELISA. Out of these people, 4 (out of 2,686 i.e., 0.14%) were females and 3 (2,263 i.e., 0.13%) were males, which indicates 57.1% and 42.8% among all the infected cases, respectively. The mean age for HCV Ab positive subjects was 50.29±10.35 years. Thus, the frequency of HCV Ab positive cases by ELISA was 0.14%, [95%CI (0.25-5.02)]. All HCV Ab-positive cases were tested by HCV RNA. The results showed that no patient was positive for HCV RNA.

**Table 1:** Some Demographic data of the study participants (N=4949)

**Variables**	**No. (%)**
Female	2686 (54.3)
Married groups	4621 (93.4)
History of surgery	2931 (59.2)
History of hospitalization	3842 (77.6)
History of blood transfusion	318 (6.4)
Drug addiction	123 (2.5)
Dental procedures	2560 (51.7)
Traveling abroad	2103 (42.5)
Abnormal urine test	1485 (30)

The highest frequency of HCV Ab was observed in the age group of 40-50 years (0.16%), followed by 50-60 and 60-70 age groups. In males, the highest frequency of anti HCV was seen in the age group less than 40 years (0.23%), and in females the age group of 60-70 years (0.3%). The frequency in the males of 60-70 age group and females less than 40 years was zero. The rate of HCV Ab in the married group was 0.15%. Regarding the level of education, the highest frequency of HCV infection in those with university degree was 0.62% and in the group with primary education was zero. In this study, the risk factors for hepatitis C infection were evaluated in all participants. The major risk factor was hospitalization (77.6%) followed by the history of surgery, dental procedures, occupation and history of blood transfusion. 

**Table 2:** Comparsion of the effects of different variables and main risk factors in anti HCV positive patients (N=4949)

Variable		Anti-HCV Ab£ ELISA¥, No. (%)		P value* (Chi-Square)	Crude OR (95% CI)
		**Positive**	**Negative**		
Gender	Male	3(0.13)	2260 (99.87)	0.87	1.22 (0.27-5.48)
	Female	4(0.14)	2682 (99.86)		
Marital Status	Single	0(0)	76 (100)	0.91	0.54(0.03-11.00)
	Married	7(0.15)	4614 (99.85)		
Age (Year)	<40	1(0.09)	1039 (99.91)	0.9	Ref
	40-50	3(0.16)	1823 (99.84)		1.71(0.17-16.45)
	50-60	2(0.14)	1406 (99.86)		1.47(0.13-16.32)
	> 60	1(0.14)	674 (99.86)		1.54(0.09-24.68)
Educational level	Illiterate	2(0.18)	1094 (99.82)	0.74	Ref
	Elementary	0(0)	1349 (100)		-
	Mid school	2(0.24)	813 (99.76)		1.34(0.18-9.57)
	High school	1(0.11)	842 (99.89)		0.65(0.06-7.17)
	University	2(0.62)	844 (99.38)		1.29(0.18-9.22)
Occupation	Employed	4(0.20)	2439 (99.80)	0.68	1.36 (0.30-6.12)
	None	3(0.11)	2503 (99.89)		
History of surgery	Yes	2(0.06)	2929 (99.94)	0.09	0.27 (0.05-1.41)
	No	5(0.25)	2013(99.75)		
History of hospitalization	Yes	3(0.07)	3839 (99.93)	0.02	0.21 (0.48-0.96)
	No	4(0.36)	1103(99.64)		
History of blood transfusion	Yes	0(0)	318 (100)	0.48	-
	No	7(0.15)	4624(99.85)		
Drug addiction	Yes	1(0.80)	122 (99.20)	0.16	6.58 (0.78-55.11)
	No	6(0.12)	4820(99.88)		
Dental procedures	Yes	2(0.10)	2558 (99.90)	0.22	0.37 (0.07-1.92)
	No	5(0.20)	2384(99.80)		
Traveling abroad	Yes	4(0.19)	2099 (99.81)	0.43	1.80 (0.40-8.07)
	No	3(0.10)	2843(99.90)		
Abnormal urine test	Yes	5(0.33)	1480 (99.67)	0.01	5.84 (1.13-30.17)
	No	2(0.05)	3462(99.95)		

£ Anti hepatitis C virus antibody.

¥ Enzyme-linked immunosorbent assay.

* P-value <0.05 was statistically significant.

Table 2 shows comparison of the effects of different variables and main risk factors in anti-HCV-positive patients. The history of hospitalization (P=0.02) and abnormal urine (P=0.01) was associated with the frequency of HCV infection. The history of hospitalization [Crude OR= 0.21, 95%CI (0.48 - 0.96)] and history of abnormal urine [Crude OR=5.84, 95% CI (1.13-30.17)] had a statistical significant relationship with the frequency of HCV infection. There was no association between HCV Ab and family history of hepatitis, history of surgery, blood transfusion, dental procedures and occupation.

## Discussion

This research project is the first epidemiological study in which the seroprevalence of HCV infection and its related risk factors has been investigated among the general population in the age group of 35-70 years. The study was performed as part of the Azar Cohort project between 2015 and 2016. Hepatitis C virus is a major factor for chronic liver disease and increases the progress of hepatic-cirrhosis and hepatocellular carcinoma. The incidence of HCV infection has been investigated among high risk groups in the previous studies and few population-based studies have been designed in this regard. The frequency of hepatitis C infection in different regions of Iran varies from 0.08% to 1.6% (6). The reasons for this heterogeneity might be related to such items as lifestyles, high-risk behaviors, overall quality of health services, cultural conditions, geographic dispersion of Iran and the impact of neighboring countries.

In this study, the frequency of anti-HCV was 0.14% by ELISA, which was lower than similar studies in provinces of Chahar Mahal and Bakhtiari (16), Fars (18) and cities of Amol (17), and Mashhad (19). Since this project was conducted in an area with religious background where family ties are strongly kept and there is a low level of social deprivation, the results can be considered as effective.

The frequency of HCV infection in females was higher than that of males, which is not in line with the results of the studies conducted by Moezzi et al. (16) and Zamani et al. (17) Meanwhile the results are consistent with the study of Akkaya et al. (20). This might be justified by the fact that the number of females included in this study was higher than that of males (54.3% females and 45.7% males). Also, there was no significant relationship between HCV infection and age, which is similar to the findings of the studies by Alizadeh et al. (21) and Teutsch et al. (22), but different from the results of the study by Miller et al. (23). This difference may be justified by the fact that HCV infection can be seen at any age. The highest frequency of HCV infection was found in the 40-50 year age group which is in line with the results of the studies by Moezzi et al. (16) and Shakeri et al. (19), though it differs from the findings of Fattahi et al. (18). The probable differences in lifestyles, ethnic groups, population density and community education about hepatitis C virus might justify the source of such contradictions.

The frequency of anti-HCV in the married group was 0.15%, which is higher than single individuals. This is also consistent with the study by Qureshi et al. (24). Regarding the level of education, in the present study, there was no significant relationship between education level and HCV infection. This finding was also found in the study of Alizade et al. (21). But there was a significant correlation between education level and HCV in the study of Todd et al (25), which indicates that the prevalence of anti HCV has been higher among those with lower education levels, which contradicts with our study. Assessing the risk factors for HCV infection in participants was another objective of this study. There was a significant relationship between the risk factors and factors influencing the transmission of the disease including hospitalization history and urine color changes. This is also consistent with the results of other studies (26, 27, 28). According to the study by Merat et al., the two factors of imprisonment and drug abuse were associated with HCV infection (10).

In a study performed by Zamani et al. non-sterile piercing and a history of first-degree affected relatives were identified as the major risk factors (17). Therefore, designing appropriate plans, screening for the acute disease, finding the affected people along with treating and controlling them should be seriously considered in the national plan in an attempt to eradicate the hepatitis C.

Certain patients, such as those with thalassemia, hemophilia and those who undergo dialysis, are also the major groups of known patients due to their frequent needs for blood transfusions and blood products. Due to the careful control of blood donations, the chance of getting infected with hepatitis C has decreased significantly in the recent years. One of the reasons for the low spread of hepatitis C in Iran is the implementation of social programs to reduce the harm caused by addiction in the country. The limitations of this study included the absence of some high-risk behaviors and risk factors for hepatitis C, along with the recall bias of being exposed to some of the possible risk factors. The participants of this study were enrolled from Azar Cohort project with the age range of 35-70. We should mention that considering the age groups less than 35 years could have altered the results of this study.

In conclusion, the following measurements should be fulfilled to reduce the incidence of hepatitis C: a comprehensive analysis of all the risk factors for hepatitis C along with a comprehensive definition of all the risk factors, increasing public awareness on the transmission routes, risk reduction strategies in high risk groups, providing free laboratory services for the community, modifying the information gathering system.
